# Smart Shear-Thinning Hydrogels as Injectable Drug Delivery Systems

**DOI:** 10.3390/polym10121317

**Published:** 2018-11-28

**Authors:** Sadaf Samimi Gharaie, Seyed Mohammad Hossein Dabiri, Mohsen Akbari

**Affiliations:** 1 Laboratory for Innovations in Microengineering (LiME), Department of Mechanical Engineering, University of Victoria, Victoria, BC V8P 5C2, Canada; s.f.sadaf@gmail.com (S.S.G.); mohammadhossein.dabiri@gmail.com (S.M.H.D.); 2 Center for Biomedical Research, University of Victoria, Victoria, BC V8P 5C2, Canada; 3 Center for Advanced Materials and Related Technologies, University of Victoria, Victoria, BC V8P 5C2, Canada; 4 Department of Informatics, Bioengineering, Robotics, and System Engineering, University of Genoa, Genoa 16145, Italy

**Keywords:** shear-thinning, hydrogels, pH-responsive, gelatin, laponite, chitosan, *N*-isopropylacrylamide, nanocomposite

## Abstract

In this study, we fabricated and characterized a smart shear-thinning hydrogel composed of gelatin and laponite for localized drug delivery. We added chitosan (Chi) and poly *N*-isopropylacrylamide-*co*-Acrylic acid (PNIPAM) particles to the shear-thinning gel to render it pH-responsive. The effects of total solid weight and the percentage of laponite in a solid mass on the rheological behavior and mechanical properties were investigated to obtain the optimum formulation. The nanocomposite gel and particles were characterized using Fourier-transform infrared spectroscopy (FTIR), scanning electron microscope (SEM), zeta potential, and dynamic light scattering techniques. Finally, release related experiment including degradability, swelling and Rhodamine B (Rd) release at various pH were performed. The results suggest that incorporation of silicate nanoplatelets in the gelatin led to the formation of the tunable porous composite, with a microstructure that was affected by introducing particles. Besides, the optimum formulation possessed shear-thinning properties with modified rheological and mechanical properties which preserved its mechanical properties while incubated in physiological conditions. The release related experiments showed that the shear-thinning materials offer pH-sensitive behavior so that the highest swelling ratio, degradation rate, and Rd release were obtained at pH 9.18. Therefore, this nanocomposite gel can be potentially used to develop pH-sensitive systems.

## 1. Introduction

Hydrogels are three-dimensional (3D) networks of hydrophilic polymers that can absorb water up to thousands times of their dry weight while preserving their structure [1]. They are formed by crosslinking polymer chains, using ionic bonds, covalent bonds, hydrogen bonds, and van der Waals interactions [2]. Hydrogels have been widely used in biomedical researches as cell culture substrates and drug depots for tissue engineering and delivery of therapeutic applications [3,4,5,6]. In addition, they are potentially able to have environmental stimuli-responsive properties so that they can undergo a volumetric phase transition in response to pH or ion changes in the environment [3]. Recently, injectable hydrogels have received significant attention because they can be delivered to the site of interest using minimally-invasive approaches.

Generally, the initial precursor solution of these hydrogels is injected into the desired area and then crosslinked in situ using ultraviolet (UV) light [7], enzymes [8], ions [9], or temperature [10]. Targeted delivery of therapeutic molecules to the desired area with injectable hydrogels can be carried out by incorporating these agents into the precursor solution. However, it is possible that the bioactive agents incorporated in an injectable hydrogel being damaged by exposing them to toxic agents or inducing non-physiological conditions [11,12]. For instance, 2-hydroxy-1-[4-(2-hydroxyethoxy) phenyl]-2-methyl-1-propanone-1-one (Irgacure 2959), a typical photoinitiator used for UV crosslinking of photocrosslinkable hydrogels, showed high cytotoxicity even at low dosages to human aortic smooth muscle cells [13]. Moreover, in situ crosslinking of the hydrogels can either result in the leakage of initial precursor solution to the adjacent tissue or blood stream, or blockage of the catheters due to premature polymerization [11,14].

To circumvent the problems inherent in in-situ forming hydrogels, shear-thinning gels have been engineered such that their viscosity reduces under higher shear rates. As such, these gels deform easier through needles and catheters and rapidly retain their original form after the removal of the mechanical force. This restoration to solid gel minimizes the limitations associated with typical injectable hydrogels. In addition, bioactive molecules such as drugs and cells can be incorporated into the main preformed hydrogel and delivered to the injection site and remain in the location as the gel recovers [12,15,16]. Recently, we engineered a shear-thinning biomaterial by blending silicate nanoplatelets and gelatin, and used as an embolic agent for endovascular embolization procedures [17]. The feasibility of using this material for endovascular embolization was demonstrated with murine and porcine in-vivo models.

In this study, we engineer a smart injectable material by incorporating pH-responsive microgels within our previously developed shear-thinning gel. The proposed gel can be deployed to the site of interest using a needle and can deliver therapeutic agents in response to local pH variations. We chose pH as an external stimulus as it is an important indicator of disease progression and can be correlated to angiogenesis, protease activity, and bacterial infection [18]. We develop a nanocomposite of gelatin and laponite loaded with Poly(*N*-isopropylacrylamide)-*co*-Acrylic acid (PNIPAM-*co*-AA) or Chi that are known as pH-sensitive drug carriers. Laponite is a charged synthetic silicate nanoplatelet (20–30 nm in diameter and 1 nm in thickness) in which the charges are distributed anisotropically so that the top and bottom surfaces possess negative charges while the positive charges are distributed along the edges [19]. On the other hand, the polymeric chain of gelatin has negatively and positively charged regions which enable intense interaction between these regions and sites of laponite with opposite charges [20]. These interactions lead to the fabrication of gelatin/laponite nanocomposite. We investigated the shear-thinning behaviour of different ratios of gelatin and laponite by performing rheological and mechanical tests to optimize the formulation of the fabricated nanocomposite. Then, positively charged Chi particles or negatively charged PNIPAM-co-AA particles were incorporated into the composite to study drug release behaviour of the gel at three different pH.

## 2. Materials and Methods

### 2.1. Materials

Gelatin type A obtained from porcine skin, chitosan (448877-medium molecular weight (190,000–310,000 Da) and deacetylation degree of 75–85%), N-isopropylacrylamide monomers (NIPAM), acrylic acid (AA), *N*,*N*′-methylenebisacrylamide (BIS), ammonium persulfate (APS), sodium dodecyl sulfate (SDS), Span 80, glutaraldehyde, Rhodamine B (Rd) and PBS tablets were purchased from Sigma-Aldrich (St. Louis, MI, USA). Synthetic silicate nanoplatelets (Laponite XLG) were obtained from Southern Clay Products, Inc. (Louisville, KY, USA). The rest of materials were provided by the following companies: sodium acetate (Bio Basic, Markham, ON, Canada), Heavy mineral oil and n-hexane (Fisher Scientific, Pittsburgh, PA, USA), ethanol (Commercial Alcohols, Toronto, ON, Canada), acetic acid and toluene (VWR, Radnor, PA, USA), and dialysis bags (Spectrum Lab, Rancho Dominguez, CA, USA).

### 2.2. Shear Thinning Gel Fabrication

A stock solution of 18% (*w*/*w*) gelatin was prepared by dissolving gelatin in Milli-Q water and heating to 40 °C to ensure that the gelatin was completely dissolved. A 9% (*w*/*w*) laponite stock solution was made in 4 °C water to prevent gelation of laponite nanoplatelets and ensure complete dissolution of the nanoclay particles. Preparation of different formulations of shear-thinning gel, as listed in Table 1, were carried out by vortex of predefined amounts of gelatin and laponite stock solutions with Milli-Q water at 3000 rpm for 5 min to obtain the desired total solid concentrations and laponite loading. This was followed by reheating the obtained gel and vortex for better dispersion of nanoplatelets. The shear-thinning gels were then stored at 4 °C. In order to prepare shear-thinning gel loaded with pH sensitive particles, particles in the amount of 1 wt % of gel were suspended in Milli-Q water. Then, the suspension was mixed with gelatin and laponite solutions through vortex as explained before. The rest of procedure was similar to the preparation of gelatin/laponite nanocomposite.

### 2.3. PNIPAM-co-AA Synthetization

A 97 mL aqueous solution containing 10 mmol of NIPAM, 0.20 mmol of BIS, 0.1 mmol of AA and 0.12 mmol of SDS was prepared and transferred to a 3-necked, RB flask. The solution was continuously purged with nitrogen during heating to polymerization temperature (70 °C). Polymerization was initiated as 3.0 mL of aqueous solution containing 0.10 mmol of APS was injected into the continuously stirring solution and allowed to react for 5 h. Purification of PNIPAM-*co*-AA was carried out by dialyzing against deionized water for 1 week with a daily water change and lyophilizing the solution using Freezemobile Virtis lyophilizer.

### 2.4. Chi Fabrication

An aqueous solution of Chi as the water phase was prepared through dissolving 1.5 wt % of Chi and 0.9 wt % of NaCl in acetic acid 1% (*v*/*v*). Then, it was emulsified by dripping the mixture into a continuous phase (oil phase) composed of mineral oil and 5% (*v*/*v*) span 80 and homogenizing at 1000 rpm for 30 min. In order to crosslink Chi, a chemical crosslinking agent (Glutaraldehyde Saturated Toluene) was added dropwise into to the Water/Oil mixture, while agitating at 300 rpm. After one hour, the mixture was centrifuged at 2000× *g* for 5 min to separate particles from the oil. The particles were further washed with hexane, ethanol 50% containing 1% Tween 20, and distilled water (5 times). Finally, the particles were lyophilized.

### 2.5. Rheological and Mechanical Analysis

Rheology and stress recovery tests were carried out based on a previously published protocol [20]. Variations of shear stress were recorded over the shear rate sweeps from 0.001 to 10 S^−1^ at room temperature for samples with different total solid weight (3, 6, and 9%). Similar rheology tests along with nondestructive mechanical analysis was performed to optimize the content of laponite in the gel. The mechanical test was conducted using ElastoSensTM Bio2 (Rheolution Instruments, Montreal, QC, Canada) to obtain the storage modulus (G′) and loss modulus (G″) at 37 °C. Injectability was further performed on the optimized content of gelatin and laponite as well as microgel loaded nanocomposite by employing a recovery analysis in which the nanocomposite was subjected to high strain rate (100%) followed by low (1%) strain rate at 1 Hz for 5 min.

### 2.6. Zeta Potential and Particle Size

Chi and PNIPAM particles, with a mixture of gelatin and laponite, were suspended in acidic, neutral, and basic media to obtain the zeta potential and hydrodynamic dimension of particles. Each sample was diluted to 0.2 wt % and vortexed for 5 min before conducting the experiments to decrease the viscosity and facilitate charge measurements. A Brookhaven BI-ZR3 Zeta Potential Analyzer (Brookhaven, Upton, NY, USA) with a 660 nm wavelength laser was used to determine the zeta potential and hydro dynamic dimensions of the samples at room temperature.

### 2.7. Scanning Electron Microscope (SEM)

SEM images were taken to study the microstructure of the shear-thinning gel and determine the effects of the introduction of particles on the microstructure of nanocomposite. Samples were freeze-dried at −80 °C and then coated with gold prior to imaging on a Hitachi S-4800 FESEM microscope (Hitachi, Tokyo, Japan) at an accelerating voltage of 1 kV.

### 2.8. Fourier-Transform Infrared Spectroscopy (FTIR)

A PerkinElmer Spectrum Two Fourier transform infrared spectrometer (PerkinElmer, Waltham, MA, USA) was used to obtain FTIR spectra by Potassium Bromide (KBr) technique. Freeze-dried samples were grounded and mixed with KBr powder to investigate the formation of nanocomposites and determine the interaction between the components. The spectra were collected within the range of 400–4000 cm^−1^.

### 2.9. Degradation and Swelling Studies

The degradation rate of the shear-thinning gel was carried out by placing an accurately weighted amount of gel in an Eppendorf tube and soaking it in buffer solutions with pH 5, 7.4, and 9.18 at 37 °C. At predetermined time intervals, the gels were centrifuged to remove the supernatant and reweigh the gel. The degradation rate was measured based on the following equation:

(1) $$\mathrm{Remaining}\;\mathrm{Weight}\;\%=\frac{Wt}{Wi}\times 100$$

where *W*_t_ is the instant weight of sample and W_i_ is the initial weight.

The swelling experiment was performed by immersing accurately weighted freeze dried shear-thinning gel in a buffer solution with different pH values. At a certain time point, the samples were removed from the media and weighted. The swelling rate was calculated as:

(2) $$\mathrm{The}\;\mathrm{degree}\;\mathrm{of}\;\mathrm{swelling}\;\%=\frac{Ws-Wd}{Wd}\times 100$$

where *W*_s_ is the weight of swollen gel and *W*_d_ is the weight of dry gel.

### 2.10. Loading Rd

30 mg of lyophilized PNIPAM-*co*-AA was immersed in a 0.1 mg/mL solution of Rd and stored in a fridge for 24 h to uptake Rd. After that, the mixture was centrifuged and washed three times with distilled water. Rd entrapment efficiency was calculated according to the following equation:

(3) $$\mathrm{EE}\;\%=\frac{Ci-Cw}{Ci}\times 100$$

where *C*_i_ is the initial concentration of Rd and *C*_w_ is the concentration of Rd in the washing supernatants.

Loading Rd on Chi particles was carried out through incorporating Rd in the initial Chi solution so that the weight ratio of Rd to Chi was 0.01. The rest of steps was as of Chi particle preparation. The entrapment efficiency was determined by immersing and shaking the predefined weight of Chi particles in acetic acid 3% (*v*/*v*). After 24 h, the suspension was sonicated for 30 min and centrifuged. Rd entrapment efficiency was obtained using the following equation:

(4) $$\mathrm{EE}\;\%=\frac{Ca}{Ct}\times 100$$

where *C*_a_ is the concentration of Rd in the aliquots and *C*_t_ is the theoretical concentration of Rd.

### 2.11. In-Vitro Drug Release

0.1 g of different loaded gel formulations were incubated in 1 mL of pH 5, 7.4, and 9.18 buffer solutions to investigate the pH sensitivity of fabricated shear-thinning gels. At predetermined time intervals, samples were centrifuged and the release media was replaced by 1 mL of buffer with the same pH to maintain a constant volume of release medium. The fluorescent intensity of the withdrawn media at each time point was measured with a Tecan infinite 200 pro plate reader (*λ*_ex_ 543 nm and *λ*_em_ 580 nm). The obtained fluorescent intensity was converted to concentration (μg/mL) using a calibration curve. The release experiment was done in triplicate.

### 2.12. Statistical Analysis

The data was presented as mean ± standard deviation. In addition, statistical analysis was carried out using One-way ANOVA with Turkey’s Honest Significant Difference (Turkey HSD) post hoc test by SPSS statistics 23 software. *p* < 0.05 was reported as significant for all statistical tests (* *p* < 0.05, ** *p* < 0.01, *** *p* < 0.001).

## 3. Results

### 3.1. Fabrication and Particle Characterization

**Figure 1:**
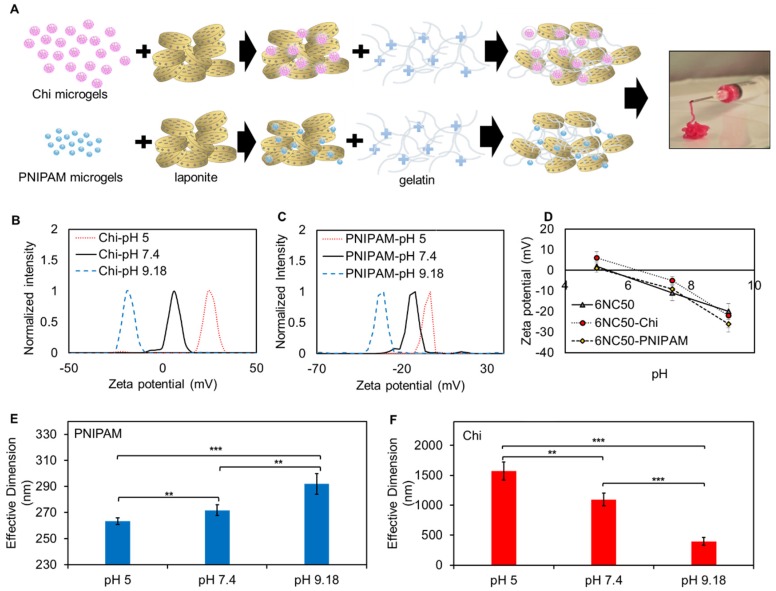
Electrostatic interactions between gelatin and laponite resulted in formation of pH responsive nanocomposite. (**A**) schematic of fabrication of shear-thinning gel containing pH responsive microgels (**B**) zeta potential of Chi particles at different pH; (**C**) zeta potential of PNIPAM-*co*-Acrylic acid (PNIPAM) particles at different pH; (**D**) zeta potential of 6NC50 and 6NC50 loaded with Chi and PNIPAM particles at different pH; (**E**) effective diameter of PNIPAM particles at different pH; (**F**) effective hydrodynamic dimension of Chi particles at different pH.

Figure 1A illustrates the schematic of the method employed to fabricate pH-responsive shear-thinning gel. The gelation process was carried out by the physical mixing of synthetic silicate nanoplatelets with porcine gelatin (Type A). Briefly, laponite was suspended in ultrapure water (Mili-Q) using a shaker which caused an enhancement in nanoparticles surface area and subsequently better interaction with gelatin. Then, gelatin stock solution was added to the prepared silicate suspension under vigorous agitation to prevent agglomeration of laponite nanoplatelets. The solution became a turbid, gel-like mixture within a few minutes due to physical crosslinking of the shear-thinning particles through an intensive electrostatic interaction between gelatin and laponite. This can be attributed to the interaction between the negatively charged sides of silicate nanoplatelets and positive sides of gelatin chain. Different compositions of shear-thinning hydrogel composites are labelled as xNCy (Table 1) where x is the total solid weight percent% (*w*/*v*) and y represents the percentage of laponite in solid mass% (*w*/*w*).

Since gelatin is a polyampholyte composed of carboxyl and amino group, its electrical charge is highly influenced by variations in pH. It has been reported that gelatin’s zeta potential constantly decreases from positive to negative values in response to a pH increase from 2–10, and reached its isoelectric point at pH = 8.3 [21]. On the other hand, laponite‘s zeta potential is always negative in this pH range and decreases with an increase in the pH [22]. Therefore, there is an electrostatic attraction between gelatin and the silicate nanoplatelets within the range of 2–8.3 and repulsion above this range. This will enable the shear-thinning gel to act as a pH-responsive carrier for the release of therapeutic agents.

These agents can be directly incorporated into initial silicate suspension/gelatin solution or they can be entrapped into different carriers and incorporated into each of the primary components. In this work, we load Rd as a drug model on PNIPAM-*co*-Acrylic acid and Chi, both of which have been extensively studied as a pH-sensitive drug carriers [23,24,25,26]. The loaded particles were suspended in Milli-Q water and dispersed in the shear-thinning gel by adding the suspension to the gelatin/laponite mixture followed by vigorous agitation.

The size and zeta potential variations of Chi and PNIPAM-*co*-AA particles in response to pH variations are shown in Figure 1B–F. It has been reported that zeta potential of Chi particles is influenced by molecular weight of Chi, the ratio between Chi and the crosslinking agent, concentration of Chi, pH, and measurement medium [27,28]. In this study, we investigated size and zeta potential changes as a function of pH in residing aqueous environment. The hydrodynamic size and zeta potential of Chi particles followed a descending trend as the pH increased. In Acidic pH, the amine group in the back bone of Chi became protonated (NH_3_^+^) and caused zeta potential to become positive. In addition, the increased hydrodynamic particle size can be attributed to the repulsion between NH_3_^+^ which facilitated transportation of water from surrounding media inside the particles. As the pH increased from the pKa of Chi (≈6.5), it was deprotonated and its zeta potential showed nearly neutral value, at pH 7.4 [29,30]. A subsequent decrease in particle size and zeta potential to 328.2 nm and −20 mv was observed when pH value of solution increased to 9.18 (Figure 1B,F). It has been shown that Chi particles reached the isoelectric point within the range of 7–7.4 [31]. In addition, Shu et al. [32] showed that ionization of amine group of Chi was highly influenced by pH so that a sharp decrease in degree of ionization was observed as the pH value of solution increased above 6. The zeta potential trend in this study is consistent with previous published works in which zeta potential of Chi particles continuously decreased with pH increase and reached to negative values in alkaline pH [27,31].

On the other hand, PNIPAM is the most frequently used thermosensitive polymer with lower critical solution temperature (LCST) around 32 °C. It has been reported that pure NIPAM has a low density of ionizable functional groups, which are essential in pH-responsive materials. Copolymerizing PNIPAM with acrylic acid not only increases the LCST to higher temperatures, but also induces pH-responsive behaviour to PNIPAM owing to its carboxylic acid group [33]. In order to simplify the text, PNIPAM-*co*-AA is hereafter replaced as PNIPAM in the labeling of samples. At pH values above the pKa of acrylic acid (≈4.2), COOH deprotonates and becomes COO^−^ which induces a negative charge to the polymeric network. The obtained result of zeta potential were consistent with this phenomenon, so that it decreased from −0.23 to −12.3 and −30.9 mv as the pH increased from 5 to 7.4 and 9.18, respectively. In addition, this transportation creates an electrostatic repulsion between the adjacent carboxylate ions within the polymeric chain and subsequently increases the hydrophilicity of the particles [34]. Therefore, the enhancement observed in hydrodynamic diameter of PNIPAM when pH increased is in keeping with the theory (Figure 1E).

Zeta potential of the shear-thinning gel at different pH after incorporation particles is illustrated in Figure 1D. Zeta potentials in all formulations of gel had descending trends owing to the decrease in the zeta potential of each component. Additionally, the zeta potential of gelatin and laponite played a major role in the complex material since the difference between the curves was negligible.

#### 3.1.1. Chemical and Structural Analysis

**Figure 2:**
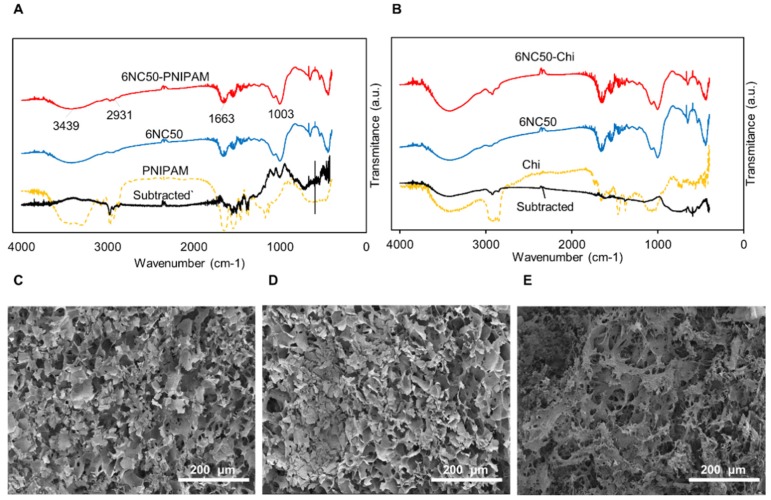
Chemical composition and microstructure of the shear-thinning gel. (**A**) FTIR spectra of 6NC50 shear-thinning gel loaded with PNIPAM particles; (**B**) FTIR spectra of 6NC50 shear-thinning gel loaded with Chi particles. SEM images illustrate the effect of adding PNIPAM and Chi particles on the microstructure of shear thinning gel (**C**) 6NC50; (**D**) 6NC50-PNIPAM; (**E**) 6NC50-Chi.

The FTIR spectra of PNIPAM, gelatin-laponite, and gelatin-laponite-PNIPAM composites were plotted in Figure 2A,B shows the FTIR spectra of Chi, gelatin-laponite, gelatin-laponite-Chi. Since a small amount of Chi and PNIPAM (1 wt % of gel) were incorporated in shear-thinning composite, the majority of the peaks were overlapped, with intense peaks for gelatin and laponite. So, subtracted spectra of [6NC50-NIPAM-6NC50] and [6NC50-Chi-6NC50] were plotted in Figure 2A,B, respectively, to compare with original spectra of PNIPAM and Chi for detecting their functional groups in the composite sample. As we can see, 6NC50 and 6NC50-NIPAM show typical bands of the main component. The stretching bands observed at 2931 and 1663 cm^−1^ correspond to C-H and C=O of carboxamide [35]. The characteristic peak related to Si-O stretching of laponite component was detected at 1003 cm^−1^. In addition, the peak appearing in the high-frequency region at 3439 cm^−1^ originated from stretching of –NH and O–H in the amide group and nanoclay [35,36]. The weak bands observed in subtracted spectra in 1650–1750 cm^−1^ arose from the carboxyl of NIPAM and acrylic acid. Moreover, the bands corresponding to –CH_3_ symmetric stretching, asymmetric –CH_2_ stretching, and –CH_3_ asymmetric stretching were detected within 2850–2975 cm^−1^ [37]. Negligible changes in peaks position along with similarity in FTIR pattern suggest that adding NIPAM to the shear-thinning gel did not remarkably affect its chemical structure. The characteristic bands of the shear-thinning gel were appeared in the spectra of gelatin-laponite-Chi, as well. Besides, the similarity between the spectra of subtracted [6NC50-Chi-6NC50] and Chi confirmed proper incorporation of Chi in the gel. As shown in the subtracted spectra, the bands related to combined peaks of OH and NH_2_ group appeared at 3416 cm^−1^. Also, the bands at 1390 and 1630 cm^−1^ correspond to the –C–O stretching of the primary alcoholic group in Chi and N-H bending, respectively [38,39]. Interestingly, the sharp band corresponding to the stretching of C-H slightly shifted to lower wavenumber, indicating some specific interaction between Chi and the gel which caused a slight decrease in the strength of the C–H bond.

Figure 2C displays SEM micrograph of fabricated gel. The gel was lyophilized before taking the image to preserve the structure and volume after water content removal. The shear-thinning gel has a porous structure with the average pore size of 74 ± 23 µm after removal of water content. Li et al. [35] stated that obtaining pore size can be tuned by introducing different ratios of gelatin to laponite. The pore size increases parallel with the increase in the gelatin component owing to the fact that gelatin has a hydrophilic functional group such as –COO^−^ and –NH_2_, which facilitate network expansion and increase the water content, therefore causing larger pore sizes. On the other hand, a more significant increase in pore size can be obtained as the concentration of laponite decreases, because it is a multifunctional crosslinker.

Figure 2D,E show SEM micrographs of the shear-thinning gel after incorporation of PNIPAM and Chi particles. Incorporating PNIPAM with negatively charged COO^−^ in shear thinning gel facilitates crosslinking, so that the average pore size slightly decreased (62 ± 16 µm). Conversely, positively charged Chi adsorbs on the top and bottom of silicate nanoplatelet and acts as a barrier in gel crosslinking. The presence of very large pores in the micrograph of the 6NC50-Chi gel is evidence for this phenomena.

#### 3.1.2. Mechanical Analysis

**Figure 3:**
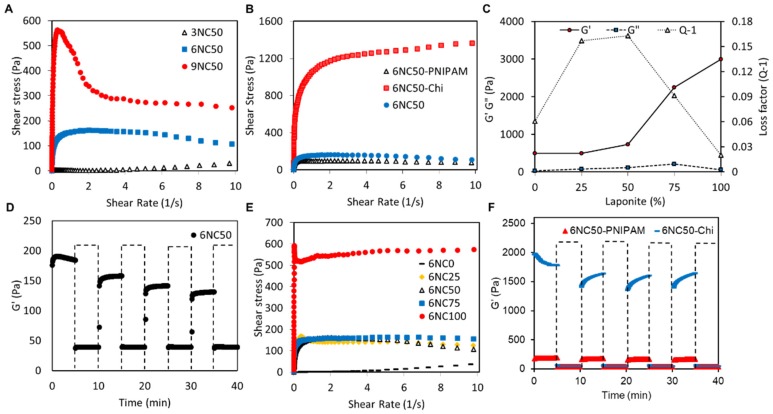
Rheological and mechanical analysis and injectability of shear-thinning hydrogel. Plot of shear stress versus shear rate as a function of (**A**) total solid weight (3NC50, 6NC50 and 9NC50) and (**B**) percentage of Laponite in solid mass (0, 25, 50, 75, and 100); (**C**) storage modulus (G′), loss modulus (G″), and loss factor of 6NCy as a function of Laponite concentration; (**D**) G′ of 6NC50 after repeated application of high strain (100% strain) and low strain (1% strain) over time (high strain condition is the regions in box); (**E**) plot of shear stress as a function of shear rate for 6NC containing pH sensitive particles; (**F**) G′ recovery of 6NC-PNIPAM and 6NC50-Chi after subjecting the gels to alternating high strain and low strain condition.

The rheological test was performed on a gelatin-laponite gel to study the shear-thinning behaviour of fabricated hydrogel. The effects of total solid weight (xNC50) and also the ratio of gelatin: laponite (6NCy) on the variation of shear stress (Pa) as a function of shear rate (1/S) were investigated. Figure 3A,B demonstrate that all the gel formulations showed shear-thinning properties. At low shear rate (10^−3^), a significant increase in shear stress was observable by increasing the total solid weight from 3 to 9 so that the shear stress enhanced from 2.4 Pa in 3NC50 to 147 and 551 Pa in 6NC50 and 9NC50, respectively. It is noteworthy that a sharp increase in the shear stress to a stable level followed by a negligible decrease at a high shear rate in 6NC50 suggests a controllable injection of this sample with respect to the other samples. Therefore, 6NCy was chosen as the optimum condition for further rheological and mechanical analysis. Incorporation of nanoclay platelets with gelatin enhanced shear stress which was due to the electrostatic interaction between negatively charged laponite and positively charged gelatin chain [21]. However, the increase in the content of laponite did not significantly affect rheological properties as the shear stress versus shear rate curve of 6NC25, 6NC50, and 6NC75 presented similar patterns. These results were consistent with previously published work [17].

The storage (*G*′) and loss (*G*″) moduli were determined at 37 °C using an ElastoSensTM Bio2 which is a non-destructive method of monitoring mechanical properties of viscoelastic materials.

The dynamic complex modulus can be obtained as shown below:

*G* = *G*′ + i*G*″ (5)

*G*′ = *G* cos δ (6)

*G*″ = *G* sin δ (7)

*G*′ represents the amount of energy stored during the loading cycle which is the indication of elastic part of the material. On the other hand, viscous part is presented by *G*″ which is the amount of dissipated energy during the loading cycle. In viscoelastic materials, the ratio between loss modulus and elastic modulus, called loss factor, refers to mechanical damping:

(8) $$Q^{-1}\;=\frac{G^{\prime \prime }}{G}=\frac{G\sin\delta }{G\cos\delta }=\tan\;\delta \;$$

where *Q*^−1^ is the loss factor and δ represents the phase difference between a dynamic stress and dynamic strain. The loss factor is being used to determine the behaviour of material so that the material has solid like behaviour as *Q*^−1^ < 1 and liquid-like behaviour when *Q*^−1^ > 1 [40].

According to Figure 3C, *G*′ is bigger than *G*″ for all samples, which indicates that all the 6NCy samples have solid-like behaviour. The storage modulus gradually increased from 492 Pa in 6NC0 to 731 Pa in 6NC50 followed by a sharp enhancement to 2246 Pa and 2994 Pa in 6NC75 and 6NC100 respectively. As shown by Figure 3C, the loss factor remarkably decreases as the content of laponite exceeds 50%, which may cause losing control of continuous injection of the shear-thinning gel. Also, the shear stress of 6NC25 sample experienced a sharp decrease when it reached its peak which may induce the same error during injecting the materials (Figure 3B). Therefore, 6NC50 was chosen to conduct further experiments.

Figure 3D demonstrates the 6NC50 stress recovery curve after repeated application of high strain shear rate (100%) and low shear strain rate (1%) over the span of 40 min. As we can see, the storage modulus remarkably decreased when high-strain shear rate was applied to the gel and it presented a liquid-like behaviour at this strain rate. While a significant increase in the storage modulus was observed at low strain rates in which gel had solid-like behaviour, the results indicated that the gel successfully recovered to solid-like behaviour after application of high strain shear rate. In addition, rheological tests along with stress-recovery analysis were carried out to examine shear-thinning properties of the gel after introducing microgels. In Figure 3E the variations of shear stress versus shear rate in 6NC50-PNIPAM was similar to that of 6NC50, indicating that shear-thinning properties were not influenced by introducing PNIPAM. In contrast, adding Chi to the gel led to a significant shift to higher shear stress. This increase can be attributed to the adsorption of Chi on the surface of laponite disks, which limits slipping of laponite nanoplatelets. Moreover, the recovery of 6NC50-PNIPAM and 6NC50-Chi after repeated application of high strain and low strain are presented in Figure 3F. The results indicate that 6NC50-PNIPAM and 6NC50-Chi recovered very fast, which is essential for avoiding unwanted flow of materials as they are injected. After three cycles of high and low strain conditions, the resulting modulus at high, 100% strain for both formulations was below 50 Pa by the end of 10 min of high strain condition, indicating rapid network disruption. After three strain cycles, a 13.5% and 17.5% decreased in storage modulus was observed in 6NC50-PNIPAM and 6NC50-Chi, respectively.

#### 3.1.3. Degradation and Swelling

**Figure 4:**
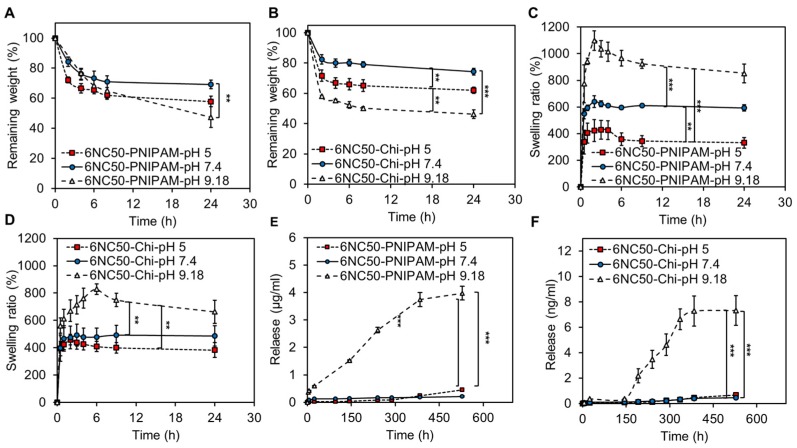
Release related experiments. Degradation, swelling, and release of Rd increases by increasing pH from 5 to 9. Degradation of (**A**) 6NC50-PNIPAM and (**B**) 6NC50-Chi at different pH; Swelling kinetics of (**C**) 6NC50-PNIPAM and (**D**) 6NC50-Chi at different pH. The highest swelling degree for both types of gel was observed in pH 9.18; cumulative Rd release from (**E**) 6NC50-PNIPAM and (**F**) 6NC50-Chi over the span of 22 days in different pH. The release was significantly higher (*** *p* < 0.001) in pH 9.18 respect to other pH.

Figure 4A,B demonstrate the degradation rate of 6NC50-PNIPAM and 6NC50-Chi in three different pH values at 37 °C within 24 h. The results showed that the main weight loss at each formulation occurred at the first 2 h. Also, both gel formulations had lower degradation rate at neutral pH so that more 70% of gels were preserved during the experiment. In addition, both formulations degrade faster in pH 9.18, and weight loss was significantly higher (*** *p* < 0.001 and ** *p* < 0.01 6NC50-Chi and 6NC50-PNIPAM, respectively) than other pH due to repulsion between the main components.

Degradation and swelling rates and their kinetics are important parameters of a drug carrier owing to their significant effects on release rate. Swelling kinetics and the ratio of 6NC50-PNIPAM and 6NC50-Chi in different pH (5, 7.4, and 9.18) are shown in Figure 4C,D. The swelling ratio was increased in both formulations by increasing pH of surrounding media so that the lowest and highest swelling ratios were observed in pH 5 and 9.18, respectively. A continuous weight gain through first 2 h of swelling time was observed in all samples, a result of the penetration of water from surrounding media, driven by osmotic pressure [41], to the porous structure of shear-thinning gel. This was followed by a slight weight decrease for the rest of swelling time in samples immersed in pH 5 and pH 7.4 buffers in which there was an electrostatic attraction between gelatin and laponite. In contrast, the highest swelling ratio of both formulations was obtained in pH 9.18 when the electrostatic repulsion between main components increased the distance between polymer chains to facilitate water penetration. In addition, shear-thinning gel exposed to pH 9.18 followed a degrading pattern after reaching to the maximum swelling ratio which can be attribute to partial dissolution of the gel in surrounding media. The difference between maximum swelling ratios of gel in pH 9.18 buffer and other buffers were statically significant for both 6NC50-PNIPAM and 6NC50-Chi (** *p* < 0.01 and *** *p* < 0.001 6NC50-Chi and 6NC50-PNIPAM, respectively). Also, there was a significant difference (** *p* < 0.01) between maximum swelling ratio of 6NC50-PNIPAM in pH 5 and 7.4 while, this difference was insignificant for 6NC50-Chi. Shear-thinning gels containing PNIPAM had higher maximum swelling ratio at every experimental conditions which can be attribute to low capability of Chi particles to uptake water in pH 7.4 and 9.18.

#### 3.1.4. Cumulative Release

Release experiments were carried out at three different pH over the course of 22 days by loading Rd with a molecular weight of 479.01 g/mol on 6NC50-PNIPAM and 6NC50-Chi. Rd is an extensively used drug model with a molecular weight similar to that of most commercial antibiotics [42]. The encapsulation efficiency (EE) of drugs on carriers can be affected by various parameters such as drug loading method, type of drug, intrinsic behaviour of carrier, particle size, and etc. [43]. Here, the drug was loaded on Chi particles through the preparation of Chi particles which resulted in very low entrapment efficiency. This low entrapment efficiency can be attributed to electrostatic repulsion between a positively charged functional group of Chi and Rd. On the other hand, a 17.9% EE was obtained as PNIPAM was immersed in Rd solution and kept at 4 °C where the particles swelling ratio was the highest, allowing Rd molecules to penetrate inside the particles. This EE is reported elsewhere [33].

Figure 4E,F show the cumulative release profile from 6NC50-PNIPAM and 6NC50-Chi, respectively. The results indicated that both shear-thinning gels had pH-responsive release profile so that they were in off-state of release at pH 5 and 7.4 and went to on-state of release at pH 9.18. As we can see, the release rate was negligible at pH 5 and 7.4 while, at pH 9.18 it was significantly higher (*** *p* < 0.001) than the other two pH. These results along with degradation results are suggesting that degradation of the shear-thinning gel was the main mechanism for the higher level of drug release at pH 9.18. As discussed before, the considerable degradation of nanocomposite gel in this condition is being attributed to the repulsion between gelatin and laponite, since both of them are negatively charged at this pH [21,22].

As be shown in Figure 4E, the release of Rd from PNIPAM particle seemed to be diffusive, since no burst release was observed at any release conditions. At pH 9.18, the release rate gradually increased to more than 3.7 (μg/mL) after 384 h followed by a slight increase for the rest of the release experiment. The major portion of entrapped Rd on the PNIPAM was released over the 22 days of an experiment, which was caused by the swelling of PNIPAM particles in pH 9.18 as evidenced by hydrodynamic size variations (Figure 1E). In contrast, 6NC50-Chi showed a significantly lower release rate compared to 6NC50-PNIPAM in pH 9.18, where the highest degradation rate was obtained for both gels. Although the gel was destroyed at pH 9.18, the Chi particles shrank and the detected 7.3 (ng/mL) Rd in media after 22 days was due to the diffusion of physically entrapped Rd. Also, low EE is another noticeable factor for the low rate of release from 6NC50-Chi.

The obtained release profiles suggest that 6NC50-PNIPAM is a better candidate for incorporation in the shear-thinning gel because it shows favourable pH responsive behaviour. In addition, the shear-thinning behavior of nanocomposite gel loaded with PNIPAM enables minimal invasive injection of the gel to the desired area for local delivery of drugs or other therapeutic agents. This is particularly useful in the treatment of wounds in which the infections are multi-strain and the pH changes significantly depending on the type of bacterial infections. This is also pertinent in the treatment of bacterial infections caused by *Pseudomonas* and *Escherichia coli* where the pH of the wound area shifts to alkaline conditions [44].

## 4. Conclusions

This study presents a smart, injectable drug delivery system, engineered by introducing Chi and PNIPAM pH responsive particles to a gelatin-laponite shear-thinning gel. Addition of PNIPAM to the nanocomposite resulted in a decrease in pore size of gel, while notably large pores were formed as Chi was incorporated within the shear-thinning gel. In addition, the injectability of the gel decreased as Chi particles were introduced to the shear-thinning nanocomposite, while PNIPAM had negligible effect on injectability. In addition, gelatin-laponite composite loaded with microgels presented desirable stress-recovery behavior so that the gel recovered very fast after shear stress removal. Furthermore, the shear-thinning gel offered the possibility of pH-responsive behaviour, since degradation and swelling rates of the gel were highly influenced by pH of surrounding media. The degradation and swelling ratios of gel in pH 9.18 buffer were significantly higher than other buffers owing to electrostatic repulsion between the main components. Besides, the pH sensitivity of the shear-thinning gel was further verified by release experiment as the release profile for both incorporated drug carriers show negligible release at acidic and neutral conditions, while the release rate notably increased in basic pH. Notably, the shear-thinning gel that contained PNIPAM particles presented better pH responsivity than the Chi one. In summary, the prepared shear-thinning nanocomposite loaded with pH responsive PNIPAM microgels presented a high potential for site delivery of therapeutic agents in which the delivery is activated in response to local basic pH.

## Figures and Tables

**Table 1:** Composition of nanocomposites.

Nanocomposite	Gelatin (g/mL)	Laponite (g/mL)	Chi (g/g)	PNIPAM (g/g)
3NC50	0.015	0.015	0.000	0.000
6NC0	0.060	0.000	0.000	0.000
6NC25	0.045	0.015	0.000	0.000
6NC50	0.030	0.030	0.000	0.000
6NC75	0.015	0.045	0.000	0.000
6NC100	0.000	0.060	0.000	0.000
9NC50	0.045	0.045	0.000	0.000
6NC50-Chi	0.030	0.030	0.010	0.000
6NC50-PNIPAM	0.030	0.030	0.000	0.010
